# Polymerized IgA Monoclonal Gammopathy Simulating Biclonal Gammopathy: A Case Report

**DOI:** 10.7759/cureus.88517

**Published:** 2025-07-22

**Authors:** Kadija Mefire, Ouissam El Mohtarim, Samira El Machtani Idrissi, Sanae Bouhsain, Abdellah Dami, Asmaa Biaz

**Affiliations:** 1 Biochemistry-Toxicology Laboratory, Faculty of Medicine and Pharmacy, Mohammed V Military Training Hospital, Mohammed V University, Rabat, MAR

**Keywords:** biclonal gammopathy, capillary electrophoresis, monoclonal gammopathy, paraprotein, polymeric immunoglobin a

## Abstract

Serum protein electrophoresis (SPEP) typically reveals a single monoclonal (M) band in cases of monoclonal gammopathy (MG). Occasionally, two bands may appear, suggesting biclonal gammopathy. However, certain monoclonal immunoglobulins (Ig), particularly IgA, may exist in multiple polymerization states, mimicking biclonal patterns. We report the case of a patient with chronic renal failure referred for routine evaluation. SPEP revealed two monoclonal bands in the beta-2 and gamma-globulin regions, raising suspicion of biclonal gammopathy. Immunotyping (IT) identified both bands as IgA lambda, suggesting either biclonality or different polymerization states of the same monoclonal protein. To clarify, serum was treated with beta-mercaptoethanol. Post-reduction IT showed persistence of the beta-2-globulin band and disappearance of the gamma-globulin band, confirming a single IgA lambda monoclonal component with two distinct polymerization states. IgA MGs can exhibit atypical electrophoretic patterns due to variable polymerization, which may mimic biclonal patterns. In such scenarios, chemical reduction with beta-mercaptoethanol serves as a critical tool for accurate diagnosis.

## Introduction

Monoclonal gammopathies (MGs) are disorders characterized by the clonal proliferation of abnormal plasma cells or lymphoplasmacytic cells, leading to the overproduction of a complete or partial monoclonal immunoglobulin (Ig) (M protein or paraprotein). The term "partial" refers to free light chains, truncated heavy chains, or other Ig fragments. Serum protein electrophoresis (SPEP) is a key diagnostic tool in the evaluation of suspected MG, typically revealing a single monoclonal band, most often in the gamma globulin region [1].

In some cases, SPEP may reveal two discrete monoclonal bands, raising suspicion for biclonal gammopathy - a condition in which two distinct M proteins are produced, either by two separate clones or, more rarely, by a single clone capable of dual secretion through class switching or dual isotype expression [2]. However, such patterns may also result from Ig polymerization, particularly of the IgA isotype, where a single monoclonal protein forms aggregates that migrate differently on electrophoresis, mimicking biclonality [3].

In these ambiguous cases, chemical reduction with agents such as β-mercaptoethanol (BME) can help differentiate between true biclonal gammopathy and polymerized monoclonal Ig. By breaking disulfide bonds, BME reduces Ig polymers into monomers, allowing more accurate interpretation of clonality on SPEP or immunotyping (IT).

This case report illustrates how IgA polymerization can mimic biclonal gammopathy on SPEP and how pretreatment with BME clarified the diagnosis, revealing a single IgA lambda clone consistent with smoldering multiple myeloma (SMM).

## Case presentation

A 73-year-old woman with a history of hypertension and long-standing chronic kidney disease (CKD) was under regular nephrology follow-up at our hospital. She remained clinically stable, with no reported symptoms such as fatigue, bone pain, recurrent infections, or anemia-related signs. As part of routine CKD monitoring, SPEP was performed using the Capillarys 3 Octa® system (Sebia®, Lisses, France) to screen for possible underlying causes of renal dysfunction.

**Figure 1:**
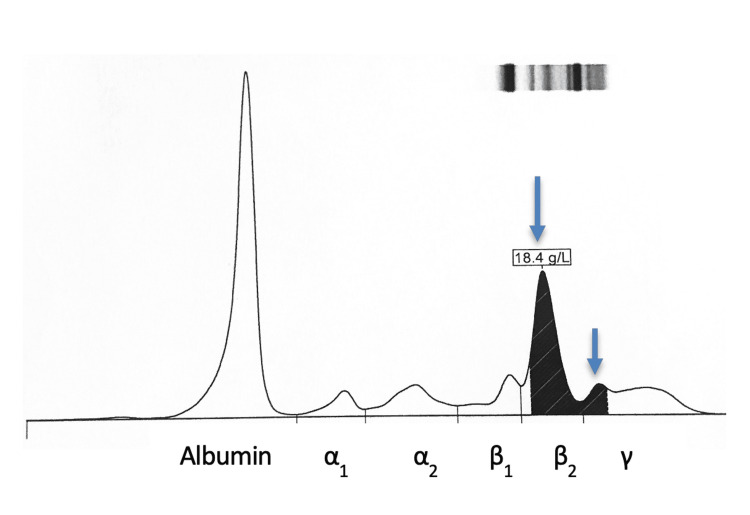
Serum protein capillary electrophoresis pattern (Capyllaris 3 Octa, Sebia®).

The SPEP revealed two discrete monoclonal bands, i.e., one in the beta-2-globulin region and another in the gamma-globulin region, with a combined concentration of 18.4 g/L (Figure 1). This electrophoretic profile initially suggested biclonal gammopathy; however, the atypical migration pattern prompted further investigation.

**Figure 2:**
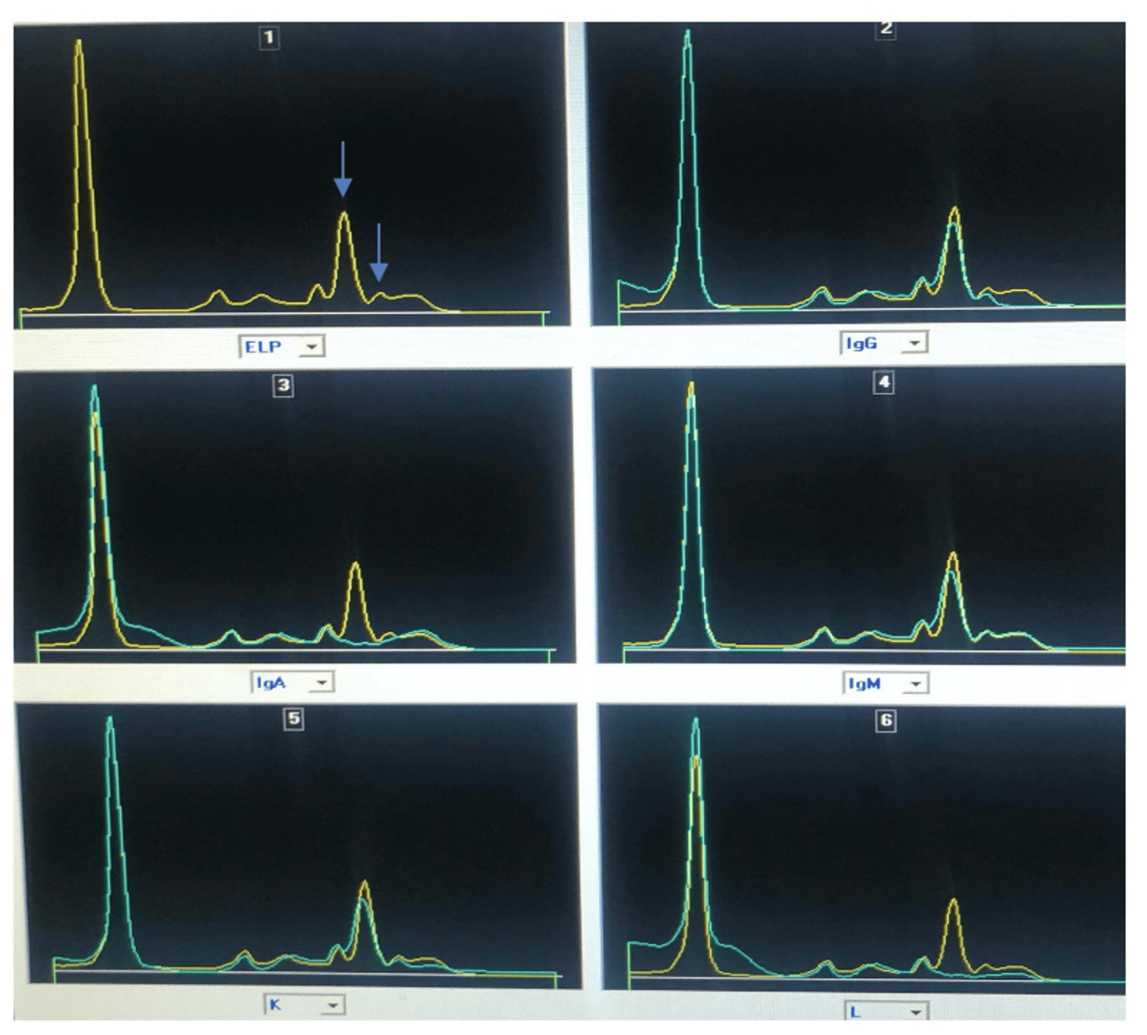
Serum immunosubtraction pattern (Capyllaris 3 Octa, Sebia ®).

IT performed on the same platform demonstrated a single broad band spanning both the IgA and lambda light chain fractions, consistent with polymerized IgA rather than two distinct monoclonal clones (Figure 2).

To confirm this hypothesis, the patient’s serum was treated with beta-mercaptoethanol (BME), a reducing agent that dissociates polymerized Ig into monomers. The reduction protocol, conducted under a chemical hood, involved preparing Solution A by mixing 90 µL distilled water with 10 µL BME (1%), followed by Solution B made by combining 10 µL of Solution A with 90 µL Fluidil. Subsequently, 300 µL of serum was incubated with Solution B at room temperature for 15 minutes. The reduced serum was then reanalyzed by IT.

**Figure 3:**
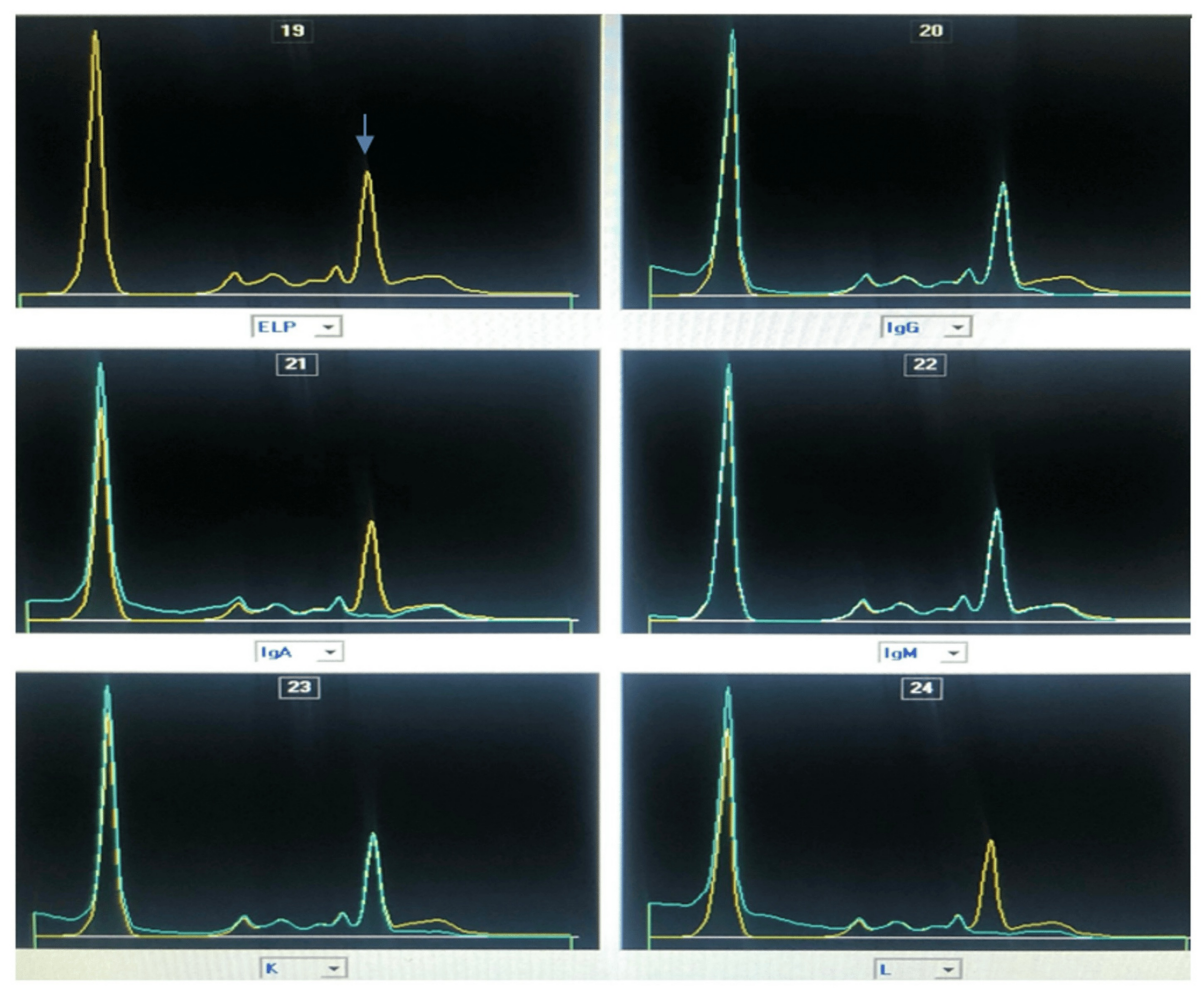
Serum immunosubtraction pattern after chemical treatment of the serum with BME (Capyllaris 3 Octa, Sebia ®).

Post-BME treatment, the two previously observed bands collapsed into a single, sharp monoclonal peak in the IgA lambda fraction, migrating in the beta-2 region (Figure 3). This confirmed that the original electrophoretic pattern was due to IgA polymerization rather than true biclonality.

Subsequent diagnostic workup confirmed the presence of a clonal plasma cell disorder. Bone marrow examination revealed 12% plasma cells. Serum-free light chain (FLC) analysis demonstrated markedly elevated FLC-λ at 635 mg/L and FLC-κ at 42 mg/L, resulting in a κ/λ ratio of 0.06, consistent with lambda light chain predominance (Table 1). Hemoglobin was mildly decreased at 11.5 g/dL, while serum calcium remained within the normal range at 95 mg/L. Whole-body MRI, including the spine and pelvis, revealed no lytic bone lesions. Renal evaluation confirmed advanced CKD, with uremia at 0.80 g/L, serum creatinine at 21 mg/L, and an estimated glomerular filtration rate (eGFR, CKD-EPI) of 23 mL/min/1.73 m² (Table 1).

**Table 1:** Hematological, biochemical, and radiological findings supporting the diagnosis of indolent multiple myeloma

Test	Result	Reference range
Hemoglobin (Hb)	11.5 g/dL	12–16 g/dL (female)
Serum calcium	95 mg/L	85–105 mg/L
Uremia	0.80 g/L	0.15–0.45 g/L
Serum creatinine	21 mg/L	6.7–11.5 mg/L
Estimated GFR (CKD-EPI)	23 mL/min/1.73 m²	>60 mL/min/1.73 m²
Serum protein electrophoresis (SPEP)	Two monoclonal bands (beta-2 and gamma regions), combined 18.4 g/L	N/A
Immunotyping (IT)	Broad polymeric band in IgA fraction; single lambda light chain band	N/A
β-mercaptoethanol reduction	Collapse to single IgA lambda peak	N/A
Serum-free light chains (FLC)	Kappa: 42 mg/L Lambda: 635 mg/L κ/λ ratio: 0.06	Kappa: 3.3–19.4 mg/L Lambda: 5.7–26.3 mg/L Ratio: 0.26–1.65
Myelogram	12% plasma cells	<5%
Renal biopsy	Chronic tubulointerstitial nephritis; no light chain or glomerular deposits	N/A

A renal biopsy demonstrated chronic tubulointerstitial nephritis without evidence of light chain deposition or glomerular involvement, suggesting that the renal pathology was not directly caused by the monoclonal protein.

Based on these findings, the patient was referred to hematology and diagnosed with smoldering multiple myeloma (SMM). This diagnosis was established by the presence of ≥10% clonal plasma cells, an abnormal free light chain ratio, and the absence of CRAB criteria (hypercalcemia, myeloma-related renal failure, anemia, or bone lesions). The patient is currently managed with nephroprotective strategies and close hematological monitoring for disease progression.

## Discussion

Monoclonal Igs are typically detected on SPEP as a sharp band in the gamma-globulin region. However, monoclonal IgM and IgA, particularly IgA, may migrate into the beta region due to their physicochemical characteristics [4]. In our patient, SPEP revealed two distinct monoclonal bands in the beta-2 and gamma regions, initially suggesting biclonal gammopathy.

Differentiating between true biclonality and polymerization-related artifacts is crucial, as misclassification may lead to overestimation of disease burden, unnecessary testing, or inappropriate therapy. In biclonal gammopathy, two distinct monoclonal proteins arise, usually from two independent plasma cell clones. Rarely, a single clone may produce two M-proteins through class-switch recombination or dual isotype expression-phenomena that, although infrequent, have been reported in the literature [2]. In other cases, especially involving IgA, a single monoclonal protein may form polymers that migrate as separate bands, mimicking biclonality [3]. This mechanism was confirmed in our patient, where reduction with BME revealed a single IgA lambda component.

Chemical reduction using BME is a valuable diagnostic tool in such situations. BME breaks disulfide bonds that link Ig monomers into dimers or polymers. This process depolymerizes the Ig, converting it into its monomeric form, which then migrates as a single, homogeneous peak on SPEP or IT [5]. In our case, BME treatment resulted in the collapse of the two bands into one, confirming IgA polymerization rather than true biclonality.

Beyond identification, polymerization has practical implications for disease monitoring. Polymerized IgA may be mistakenly quantified as two separate M-proteins, leading to inaccurate assessment of disease burden. Studies have shown that patients with polymeric IgA may present with apparently higher M-protein levels compared to those with monomeric IgA [6,7]. Moreover, IgA polymers have been associated with hyperviscosity syndrome and assay interference, such as falsely elevated calcium levels due to binding of calcium by IgA complexes [3,8]. Although our patient did not exhibit hyperviscosity or hypercalcemia, these risks underscore the importance of recognizing IgA polymerization.

While immunofixation (IFE) remains the gold standard for paraprotein characterization, it may fail to resolve co-migrating polymers or yield ambiguous results [5]. Capillary electrophoresis-based IT, especially when combined with BME pretreatment, offers clearer resolution and helps avoid misdiagnosis.

According to the International Myeloma Working Group (IMWG), SMM is defined by ≥10% clonal plasma cells in the bone marrow and/or a serum M-protein level ≥30 g/L, in the absence of CRAB criteria (hypercalcemia, renal failure related to myeloma, anemia, or bone lesions) or amyloidosis [9]. In our case, the diagnosis of SMM was supported by the presence of 12% bone marrow plasma cells and a markedly abnormal free light chain ratio, despite an IgA level <30 g/L and no myeloma-defining events. Patients with SMM require careful monitoring for progression to symptomatic multiple myeloma [10]. Our patient is currently being managed with nephroprotective therapy and close hematological monitoring for potential progression.

## Conclusions

Accurate differentiation between biclonal gammopathy and monoclonal gammopathy due to polymerized IgA is essential for appropriate clinical management and follow-up. In this case, BME pretreatment was pivotal in revising the initial suspicion of biclonal gammopathy to a diagnosis of SMM by revealing a monoclonal IgA lambda component. Misinterpretation of electrophoretic patterns can lead to overtreatment, patient anxiety, or missed opportunities to monitor disease progression.

Although polymerized IgA is relatively uncommon, the use of reducing agents like BME in serum protein electrophoresis and IT is especially valuable when atypical banding patterns are observed. While this method is cost-effective and relatively simple, it requires appropriate laboratory expertise and controlled conditions to ensure reliable interpretation.

Incorporating BME reduction selectively into diagnostic workflows enhances the precision of plasma cell disorder classification and supports more effective, individualized patient care.
